# Elucidation of the interaction of apocarotenoids with calf thymus DNA by biophysical techniques and *in vitro* study in MCF-7 cells to explore their potential in cancer therapy

**DOI:** 10.22038/IJBMS.2023.69926.15213

**Published:** 2023

**Authors:** Jill Elza Mathew, Siva Ramamoorthy

**Affiliations:** 1 School of BioSciences and Technology, Vellore Institute of Technology (VIT), Vellore 632014, Tamil Nadu, India

**Keywords:** Apocarotenoids, Cell cycle analysis, Circular dichroism, DNA binding, DNA melt curve, Groove binding, UV-visible spectroscopy, Viscosity

## Abstract

**Objective(s):**

DNA is one of the targets of cancer-therapeutic small molecules. Cisplatin, a DNA intercalator, is one of the first-line drugs in the cancer chemo regimen which comes with health-compromising side effects during chemotherapy. The synergistic effect of natural molecules with cisplatin can help to potentiate its anti-cancer efficacy and decrease its negative effect on health. Here, we report the interaction of cisplatin with calf thymus-DNA (ct-DNA) in combination with natural molecules like apocarotenoids which are reported for their therapeutic properties.

**Materials and Methods:**

The combinatorial effect of apocarotenoids on ct-DNA was explored through various biophysical techniques such as UV-Visible spectroscopy, circular dichroism studies, DNA melt curve analysis, viscosity measurements, and an *in vitro* study in MCF-7 cells by cell cycle analysis.

**Results:**

UV-Visible spectroscopy studies suggest apocarotenoids and their combination shows a non-intercalative mode of binding. Circular dichroism analysis showed no major changes in DNA form during the interaction of DNA with apocarotenoids and their respective combinations with cisplatin, which is suggestive of the groove-binding mode of apocarotenoids. DNA melt curve analysis showed a decrease in the intensity of the fluorescence for apocarotenoids with cisplatin which indicates the possibility of DNA interaction through groove binding. Viscosity studies suggested a groove binding mode of interaction of ct-DNA with apocarotenoids and their combination as there was minimal change in the viscosity measurements. The *in vitro* analysis exhibits that the apocarotenoids and their combination have a considerable effect on DNA synthesis.

**Conclusion:**

This study provides a better perspective on the possible mode of interaction between ct-DNA and natural molecules along with cisplatin.

## Introduction

The study of the interaction of DNA with different molecules is of great essence in drug design, development, and improvement (1). An in-depth understanding of the mode of interaction between drugs/ligands and DNA can provide information on gene mutation and the etiology of a disease. In cancer research, many chemotherapeutic drugs like cisplatin, mitoxantrone, etc., target DNA and affect its replication and transcription, cause DNA damage, and block cellular division which eventually leads to cell death (2). Therefore, a better perception of the multiple types of interaction between DNA and different molecules can be a useful tool for the initial screening of drug efficacy (3,4). There are multiple reports on different modes of binding interactions between DNA and molecules from natural sources such as anthraquinones, carotenoids, and flavonoids which have the potential to serve as candidate drugs for therapeutics (2, 5, 6). Natural compounds like apocarotenoids which are polyene compounds derived from the oxidative cleavage of carotenoids naturally produced in plants have been reported for their medicinal properties (7). Apocarotenoid is known to act as an effective therapeutic agent for several ailments such as asthma, skin problems, urinary infections, leprosy, neurological defects, inflammatory diseases, and cancer (8, 9). 

Bixin is a reddish-yellow apocarotenoid procured from the seeds of *Bixa orellana *L., usually known as achiote, annatto, or lipstick tree. Bixin is a major constituent (80%) of the annatto extract present in the seed aril which is responsible for the reddish-yellow color of the seed and extract (10). It is used as a spice in Latin America and as a food colorant in beverages, cosmetics, pharmaceuticals, snacks, sauces, margarine, confections, and dairy products like cheese, cream, and buttermilk deserts in different countries (11). Bixin exhibits various pharmacological activities like antifungal, antibacterial, antioxidant, antidiarrheal, anti-inflammatory, anti-tyrosinase, anti-aging, and anticancer (12, 13). Several studies have proven that bixin exhibits antitumor activity in B16 (melanoma), MCF-7 (breast), HCT-116 (colon), and HL60 (leukemia) cell lines (13-16). 

Crocin is one of the main secondary metabolites found in the dry stigma of *Crocus sativus* L., which is used as a spice. Similar to bixin, crocin also displays various therapeutic properties such as antioxidant, anti-tyrosinase, anti-inflammatory, anti-diabetic, cardio and hepatic protective properties, anti-cancer activity against cervical carcinoma (HeLa), colorectal cancer cells (HCT-116, SW-480, and HT-29), colon carcinoma (DHD/k12), and hepatocellular carcinoma (Hep G2) cell lines (12, 17).

Cisplatin has been commonly used as a first-line treatment for various types of cancer. The negative aspect of this drug is its high systemic toxicity toward healthy cells (18). Our lab has previously published work with norbixin, a bioactive molecule from *Bixa* seeds, and its interaction with ct-DNA and DNA binding studies with other natural compounds. Hence, in continuation to exploring different apocarotenoids, a combinatorial study of bixin-cisplatin and crocin-cisplatin with DNA was investigated through spectrometric, molecular, and physicochemical analysis.

## Materials and Methods

***Materials***

Sodium-calf thymus DNA (ct-DNA), crocin, and cisplatin were obtained from Sigma-Aldrich, and analytical-grade solvents were used. Bixin was extracted from *Bixa orellana *L. seeds as per Pattanaik *et al*. (2018) and purified using preparative HPLC (19). Crocin and cisplatin were dissolved in double distilled water and DMSO, respectively. The ct-DNA stock solution was made by dissolving the ct-DNA for 24 hr at 4 ^°^C in Tris-EDTA buffer (pH 7.4) to create a homogenous solution. Beer-Lambert’s equation was used to calculate the concentration of the ct-DNA solution using UV absorbance at 260 nm (molar extinction coefficient: 6600 M-1 cm-1)(20, 21). Bixin was dissolved in 1% DMSO. The baseline for all the subsequent analyses involving bixin was obtained from a 1% DMSO solution. All the chemicals for cell culture studies were obtained from HiMedia Laboratories and propidium-iodide solution from Sigma-Aldrich. TB green master mix was obtained from Takara Bio Inc. To determine the combinatorial effect of apocarotenoids(bixin and crocin) with cisplatin, seven parts of apocarotenoid were mixed with three parts of cisplatin.

***Methods***

*UV-visible spectroscopy*

To investigate the interaction between ct-DNA and apocarotenoids in the absence and presence of DNA at various concentrations, UV-visible spectra were collected. Apocarotenoids and their combination with cisplatin were kept at a fixed concentration of 100 uM and 150 uM, respectively while varying the ct-DNA concentration from 20-100 uM to study the spectral shift. A quartz cuvette with a 1 cm route length was utilized to record the spectra in the 200-800 nm range at room temperature using a Cary 3500 Multicell UV-Vis Spectrophotometer. Before each reading, the DNA sample combination was incubated for 10 min at room temperature (22, 23).

*Circular dichroism measurements*

CD spectra were captured using a JASCO (J-815) spectropolarimeter with a Peltier temperature controller at room temperature using a 1 cm rectangular quartz cuvette at a scanning rate of 100 nm/min (24). The wavelength range of 200-300 nm was considered for recording spectra, and three scans were taken for each sample which was averaged automatically. Tris-EDTA buffer (pH 7.4) which was used to dissolve DNA and apocarotenoids, was read first for baseline correction. Apocarotenoids were mixed incrementally from 100 µM to 500 µM to a constant DNA concentration of 200 µM. The mixture was kept at room temperature for an incubation period of 30 min before each reading (25). 

*DNA melt curve analysis*

cDNA synthesized from a mammalian cancer cell line (MCF-7) was used as a template for PCR amplification with a TB green master mix containing SYBR green. Human mTOR primers were used to obtain a 150 bp size of amplicon. The polymerase chain reaction conditions are as follows: -95 ^°^C for 3 min, 95 ^°^C for 10 sec, 65 ^°^C for 30 sec, and the cycle repeated 40 times, 65 ^°^C for 5 sec and 95 ^°^C for 50 sec. The following primers were used to amplify 150 bp amplicon from cDNA: Forward primer (5’-TGCCTTTGAGCAGAAAAGGT-3’) and reverse primer (5’-CGACCGCACATCATCTCGTA-3’). The amplicon was taken in equimolar concentrations, to which crocin and bixin (2.5 µM), cisplatin (0.75 µM), and their respective combination in defined proportions (7:3) were mixed in separate PCR tubes. Melt curve analysis was conducted in BioRad CT1000 thermal cycler mounted with CFX96 Real-Time system. The temperature was increased gradually at the rate of 0.5 ^°^C per second from 60 ^°^C to 100 ^°^C (26). 

*Viscosity measurements*

The change in the viscosity of 200 µM ct-DNA upon the addition of apocarotenoids and cisplatin at a constant temperature of 25 ^°^C±0.5 ^°^C was determined using Ostwald’s viscometer. A varying concentration of compound (20-100 µM) was added to the ct-DNA solution (200 µM) to obtain values of r=0.1, 0.2, 0.3, 0.4, and 0.5. The flow time (t) of the DNA-apocarotenoid or DNA-apocarotenoid-cisplatin solution through the capillary was recorded using a digital stopwatch and the mean value of the triplicate readings was used to calculate the average viscosity of the corresponding sample. The data has been represented as a graph of the relative viscosity of ct-DNA (n/n_0_)^1/3^ versus r (r=[compound]/[ct-DNA]) where n and n_0_ represent the viscosity of ct-DNA in the presence and absence of compound, respectively. Flow time correction was done using the flow for the buffer alone (t_0_). Flow time=t-t_0 _(20, 21).

*Cell cycle analysis*

The biological effect of possible interaction between apocarotenoids and DNA was tested *in vitro* in a mammalian cell line. MCF7 cells were grown to 70% confluency using Dulbecco’s Modified Eagle Media supplemented with 10% Fetal Bovine Serum. Before treating the cell with the compounds of interest, the mitotic cell cycle was synchronized using serum-deprived media (1% FBS in DMEM). The synchronized cells were treated with apocarotenoids and their combination in the range of 100-500 µM for 18 hr. Subsequently, the cells were washed in PBS and harvested with 0.25% trypsin in PBS. Harvested cells were fixed/permeabilized by adding 70% ice-cold ethanol and incubated overnight at 4-8 ^°^C. The fixed cells were treated with RNase A solution (100 µg/ml) to ensure the staining of DNA alone. Before flow cytometry analysis, propidium-iodide solution (20 g/ml) was added to the fixed cells and incubated at room temperature for 30 min. The singlet cell population was distinguished by PI-Area vs FSC-Height scatter plot and was gated for downstream analysis. The cell cycle stages of the gated population were determined by count versus PI intensity histogram plot (27).

## Results

***UV-Visible spectral studies***

**Figure 1:**
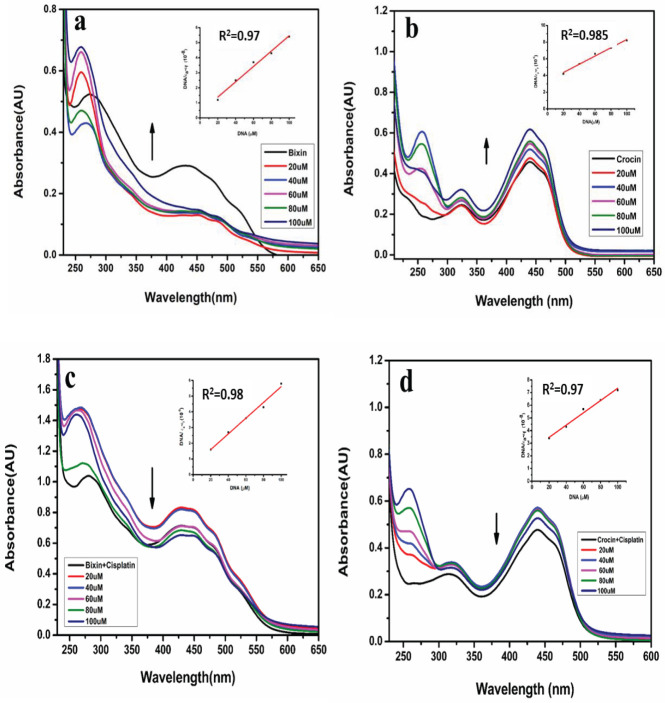
(a) UV-visible spectra of Bixin (100 µM) with ct-DNA in increasing concentrations (20-100 µM) and (b) UV-Visible spectra of crocin (100 µM) with ct-DNA in increasing concentrations (20-100 µM) (c) UV-Visible spectra of Bixin+Cisplatin (150 µM) with ct-DNA in increasing concentrations (20-100 µM) and (d) UV-Visible spectra of Crocin+Cisplatin (150 µM) with ct-DNA in increasing concentrations (20-100 µM)

An electronic spectroscopic approach was used to examine the impact of apocarotenoids and their combination with cisplatin to observe any potential spectral alterations incurred by the compound’s interaction with DNA. As illustrated in Figure 1A, bixin and its combination with cisplatin with increasing concentration of DNA show hyperchromism with a shift in the absorption peak of bixin from 432 nm to 450 nm (redshift) on the addition of DNA. This could be indicative of a partial intercalation. Similarly, in Figure 1B, crocin shows hyperchromism with increasing DNA concentration without any apparent shift in the absorption peak. On the contrary, apocarotenoids with cisplatin display hypochromism without any noticeable change in the peak as illustrated in Figures 1C and 1D which could be due to the presence of cisplatin which is an intercalator. The binding constant (K_b_) value is presented in Table 1.

***Circular dichroism spectrometry***

**Figure 2.**
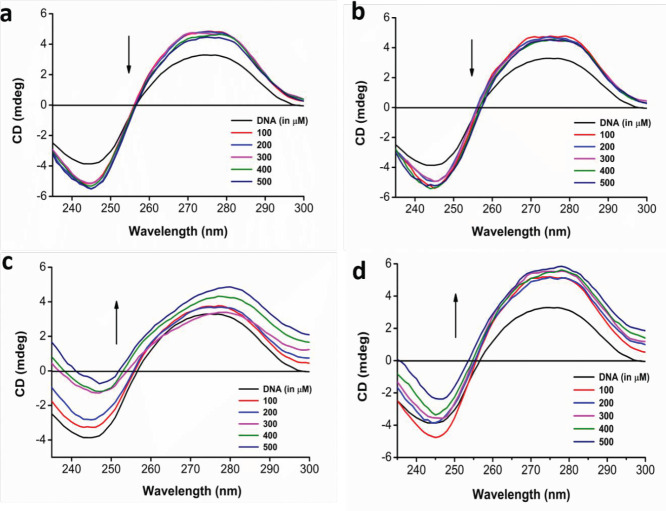
(a) CD spectra of DNA (200 µM) with Bixin in increasing concentrations (100-500 µM); (b) CD spectra of DNA (200 µM) with Bixin+Cisplatin in increasing concentrations (100-500 µM); (c) CD spectra of DNA (200 µM) with crocin in increasing concentrations (100-500 µM) and (d) CD spectra of DNA (200 µM) with Crocin+Cisplatin in increasing concentrations (100-500 µM)

Any possible effect of apocarotenoids on interaction with ct-DNA resulting in conformational changes was studied using circular dichroism spectroscopy. The spectrum obtained from all the treatments shows that the interaction between apocarotenoids does not cause any shift in the B conformation of the ct-DNA. However, the amplitude of peaks at 275 nm and 245 nm which are typical for a B-form DNA, was observed to increase in a concentration-dependent manner. As illustrated in Figures 2A and B, bixin and its combination with cisplatin in increasing concentration exhibit a decrease in peak intensities without any change in the peak position. Whereas, crocin and its combination with cisplatin in Figure 2C and D, show an increase in the amplitude of respective peaks as concentration increases. 

***DNA melt curve analysis***

**Figure 3:**
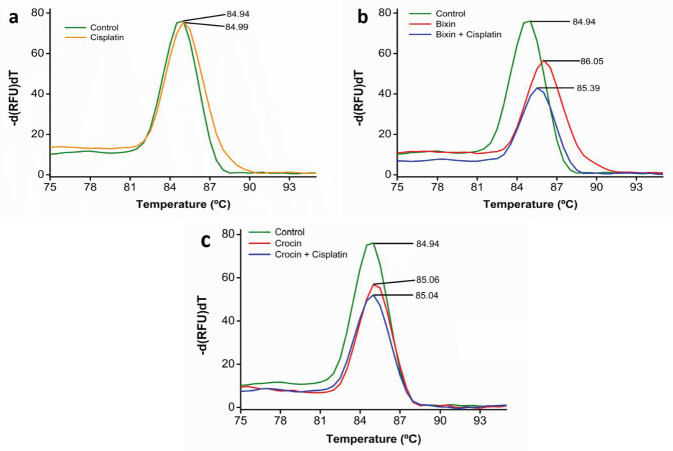
**.** (a) Represents the melt curve of the DNA sample with cisplatin; (b) and (c) Show the varying fluorescence intensities of bixin and crocin with their combinations, respectively

Binding/intercalation of extrinsic compounds to DNA should affect its strand separation or ‘melt properties’ in a typical melt curve analysis done in a Real-Time PCR. Bixin (5 µM) when added with DNA (2.5 µM) was observed to decrease the intensity of relative fluorescence and increase the melting point of DNA. The fluorescence intensity of cisplatin with DNA has been illustrated in Figure 3A. The mixture of the same with cisplatin in a 7:3 proportion (7 parts of bixin and 3 parts of cisplatin) has further decreased the fluorescence intensity with a meager change in the melting temperature as shown in Figure 3B. A similar pattern was observed in the crocin (5 µM) added tubes differing only in the magnitude of fluorescence intensity and melting temperature as shown in Figure 3C. 

***Viscosity measurements***

**Figure 4:**
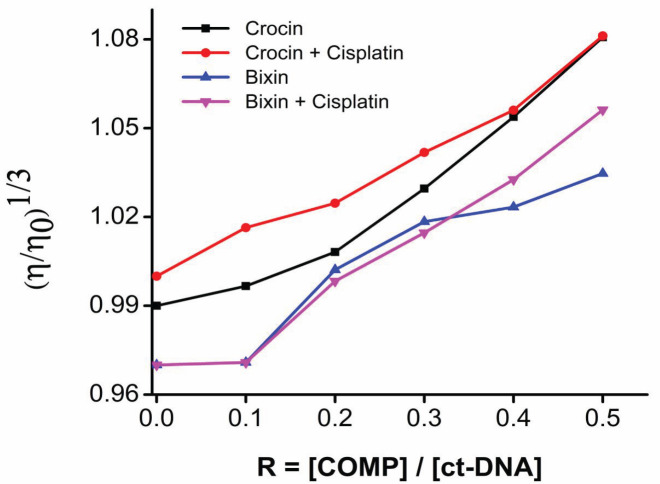
Effect of apocarotenoids and their combinations in varying concentrations (r=0.1, 0.2, 0.3, 0.4, and 0.5) on ct-DNA (200 µM)

The relative viscosities of the mixtures of ct-DNA and the compounds of interest are plotted in Figure 4. The viscosity of the solution progressively increased with increasing apocarotenoid concentration. The viscosity of the ct-DNA solution rose from 0.99 to 1.07 after the addition of crocin and crocin-cisplatin. In contrast, bixin and its combination with cisplatin increased the viscosity of the solution from 0.97, 1.02, and 1.05, respectively. 

***Cell cycle analysis***

**Figure 5:**
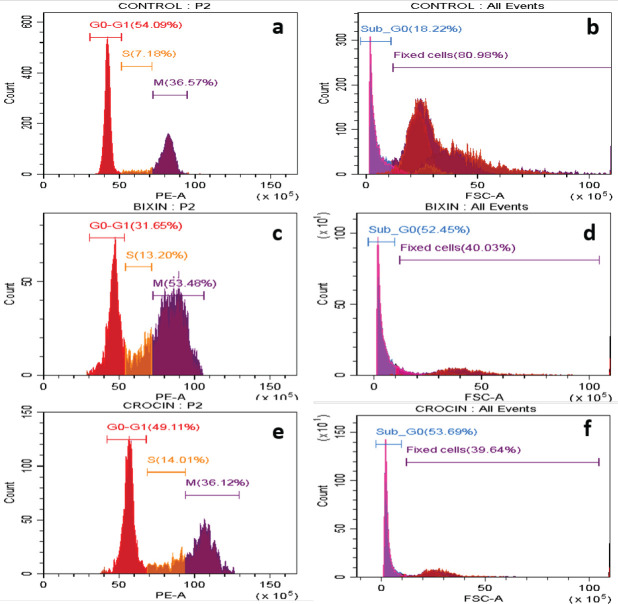
MCF-7 cells were treated with apocarotenoids and in combination with cisplatin for 18 h and stained using propidium iodide

**Figure 6:**
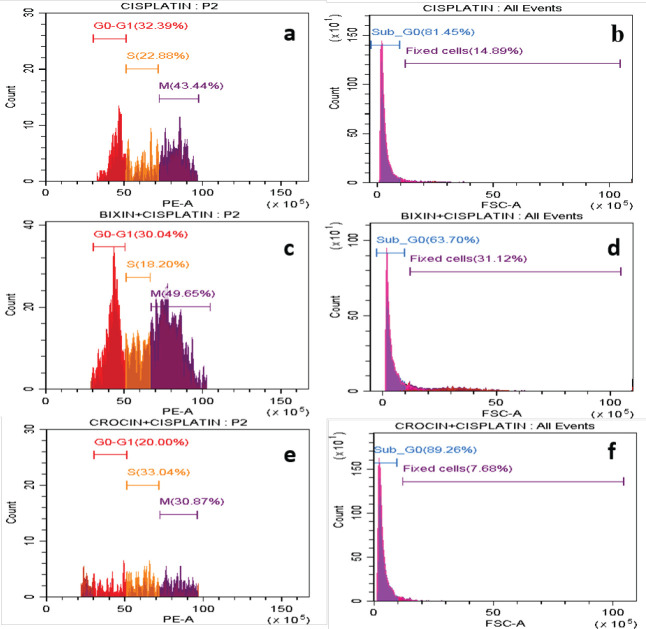
MCF-7 cells were treated with apocarotenoids in combination with cisplatin for 18 hr and stained using propidium iodide

The cells after treatment with two apocarotenoids and in combination with cisplatin were fixed and stained with propidium dye. The different phases of the cell cycle for different treatments are highlighted in Table 2. All the treated cells had a significant increase in the Sub-G0 population, remarkably in cisplatin and cisplatin administered in combination with apocarotenoids as observed in Figures 5 and 6. In addition, arrest at the S phase is observed across all the treated cells. When cisplatin was combined with apocarotenoids, the arrest at the S phase was observed to be relatively higher. In brief, the treatment with apocarotenoids or apocarotenoids and cisplatin affected arrest in aneuploidy phases (S and M phase) at the expense of the diploid phase (G0-G1) of cells.

## Discussion

***UV-visible spectroscopy***

UV-Visible spectroscopy is one of the frequently employed techniques to examine the interaction between DNA and small molecules. It helps to understand the type of interaction based on the spectral shift observed when the ligand is in its free form as well as when bound to DNA. Hypochromism and bathochromism (redshift) are typically formed as a consequence of substance/ligand binding with DNA by intercalation. Hypochromism appears due to the shortening of the distance between the intercalated substance and the DNA base, as a result, there is a decrease in the π- π* electron transition, and a redshift is observed. Hyperchromism can arise through non-covalent interactions occurring outside the DNA helix due to the electrostatic attraction to the phosphate group of the DNA backbone or due to groove binding **(**28). Partial uncoiling of the DNA helix, which exposes more DNA bases can also lead to hyperchromism as there is lower base-base interaction. Bixin and crocin exhibit hyperchromism without a significant peak shift which suggests a non-intercalative mode of binding (29**)**. Apocarotenoids in combination with cisplatin display hypochromism without any notable change in the peak which could be due to the presence of cisplatin which is a known intercalator. The binding constant value for apocarotenoids and their combination was calculated using the following equation:

[DNA]/(ε_a_-ε_f_)= [DNA]/(ε_b_-ε_f_)+1/K_b_(ε_b_-ε_f_) 

where (DNA) is the concentration of the ct-DNA taken, ε_a_, ε_f,_ and ε_b _represent the apparent extinction coefficient for the complex formed. ε_a_ corresponds to Abs/(complex) with DNA, ε_f_ corresponds to the absorbance in the absence of DNA, and ε_b_ corresponds to fully bound DNA. The slope and the intercept values were obtained from the plot of (DNA)/(ε_a_-ε_f_) vs (DNA) and the binding constant value K_b_ was obtained from the slope-to-intercept ratio (25). The K_b _value for the bixin and bixin combination acquired is 4.2×10^3^ and 8.3×10^3^, respectively and the K_b _values for the crocin and crocin combination are 1.46×10^3 ^and 1.94×10^3 ^respectively. The binding constant observed is similar to that of other compounds of natural source such as vincristine (K_b_=1×10^3^), caffeine (K_b_=9.7×10^3^), naringin (K_b_=3.1×10^3^), and morin (K_b_=5.99×10^3^). The binding constant value suggests the mode of binding to be non-intercalative in nature(30-32).

***Circular dichroism spectrometry***

Circular dichroism studies provide insight into the conformational changes of the DNA structure and help to study the binding pattern of various compounds with DNA. In typical CD spectra of ct-DNA, a positive peak is observed at 275 nm due to base stacking, with a negative peak at 245 nm due to DNA helicity, and, these peaks are characteristics of B-form DNA. But when there is groove binding or electrostatic interaction with the B-form DNA, no change or minuscule change in the amplitude or peak shift in the spectra is observed. Whereas, intercalation of molecules with B-form DNA brings about a considerable shift in the peak position and amplitude which can bring about a change from B-form to A-form DNA (20, 26). Bixin and its combination with cisplatin in increasing concentration exhibit a decrease in peak intensities at 275 nm and 245 nm without any change in the peak position. Whereas, crocin and its combination with cisplatin show an increase in the amplitude of respective peaks as concentration increases without any peak shift. The CD spectra of bixin and crocin’s interaction with ct-DNA did not show a noticeable shift in peak position in the spectra but a change in the amplitude of the B-form ‘signature’ peaks was observed. This shows that the apocarotenoids and their combination with cisplatin did not have a major effect on the helicity of the DNA at 245 nm nor did it interfere with base stacking of the DNA at 275 nm and induce any change from B-form to A-form of DNA. Hence, it is likely that the apocarotenoids and their combination interact with ct-DNA through groove binding instead of intercalation. Cisplatin, being a known DNA intercalator did not make significant shifts in the CD-spectra when used in combination with apocarotenoids (33). 

***DNA melt curve analysis***

In conventional DNA melting temperature studies, the UV measurements can provide incorrect results if the absorption peak of the compound and absorption maximum of DNA overlap. Hence melt curve analysis done by RT-PCR with extrinsic fluorescent dyes can overcome this problem (34). In the cellular environment, cisplatin or any platinum-based drug bind to DNA and prevent its opening for replication or translation. Thus, such an irreversible binding/damage to DNA forces the cell to initiate DNA repair machinery, failing which, apoptosis is triggered. Some alkylating compounds may directly damage the DNA and trigger a similar response. Whether apocarotenoids cause such groove binding/damage was tested through a typical precision melt analysis of DNA in the presence of SYBR green in RT-PCR. As anticipated, the cisplatin in the given concentration did not affect the melting point of the DNA significantly. But when both bixin and crocin were added to the same mixture, the intensity of the relative fluorescence decreased with a significant change in the melting points. In this case, it is possible that bixin and crocin through groove binding could be blocking the binding of SYBR green to double-strand DNA. Hence there is a decrease in relative fluorescence. Another possibility, that this could be due to the quenching of SYBR green’s fluorescence by the apocarotenoids themselves, could not be ruled out yet. 

***Viscosity measurements***

To further understand the interaction of apocarotenoids with DNA, viscosity measurement using Ostwald’s viscometry was executed. The viscosity measurement of DNA solution can serve as a good technique to study the change in the DNA length upon the addition of a DNA intercalating compound and could provide information on the mode of interaction between DNA and the compound. In the case of intercalation, the DNA viscosity increases as a result of the lengthening of the DNA helix. This is due to the separation of the base pair to accommodate the compound bound to the DNA which increases the length. In partial intercalation, the length of the DNA is reduced as a result of a kink or bend in the helical structure reducing the DNA viscosity. Groove binding and electrostatic mode of interaction show minimal changes in the measurement or remain unchanged (35, 36). Here, the viscosity measurements of ctDNA with apocarotenoids and their combinations display a negligible increase in the viscosity which is of groove binding or electrostatic mode of binding rather than intercalation. Hence it can be concluded that apocarotenoids and their combination with cisplatin prefer a non-intercalative mode of interaction, though the combination shows a slight increase in the viscosity of the DNA solution likely due to the presence of cisplatin. Based on the above observations, the apocarotenoids likely tend to bind to the groove of DNA but not intercalation. Hence it can be concluded that apocarotenoids and their combination with cisplatin prefer a non-interactive mode of interaction, though the combination shows a slight increase in the viscosity of the DNA solution likely due to the presence of cisplatin. Based on the above observations, the apocarotenoids likely tend to bind to the groove of DNA and do not intercalate which could be due to the lack of planar aromatic rings which can intercalate to the DNA structure (37, 38).

***Cell cycle analysis***

Given such interaction, we wanted to test if that could have any biological effect *in vitro*. As expected, cisplatin arrested almost 22% of the treated cells at the synthesis phase and the majority of the cells were dead (81.45%). Bixin, bixin combination, crocin, and crocin combination have also considerably arrested cells at the synthesis phase (13.20%, 18.20%, 14.01%, and 33.04%, respectively) as against 7.18% in the control cells. This indicates that despite the lack of DNA intercalation properties, these apocarotenoids can still affect cellular DNA synthesis. In combination with cisplatin, this effect was only exacerbated (15). Apocarotenoids and cisplatin are known for their apoptotic mode of action in cancer cells and are effectively active in the apoptotic pathways in various types of cancer (13, 14, 16). Hence it is possible that apocarotenoids and their combination which shows a higher percentage of cell death, binds to the DNA and triggers the apoptotic pathway in MCF-7 cells leading to cell death and can be a candidate drug option for combinatorial treatment which can be explored in cancer treatment regime.

**Table 1:** Shows the binding constant values for the ct-DNA-compound interaction

Compound	Binding constant (K_b_)
Bixin	4.2 ×10^3^
Bixin+Cisplatin	8.3 ×10^3^
Crocin	1.4 ×10^3^
Crocin+Cisplatin	1.9 ×10^3^

a, b represents the histogram for cell cycle phases and sub-G0 populations in control flasks respectively; c, d represents the histogram of cell cycle phases of cells treated with bixin (250 µM) and its sub-G0 population, and; e, f represents the histogram of cell cycle phases of cells treated with crocin (250 µM) and its sub-G0 population respectively

a, b represent the histogram of cell cycle phases of cells treated with cisplatin (50 µM) and its sub-G0 population; c, d represent the histogram of cell cycle phases of cells treated with bixin and cisplatin combination (250 µM+50 µM); e, f represent the histogram of cell cycle phases for crocin and cisplatin-treated cells at (250 µM+50 µM) and its sub-G0 population

**Table 2:** Shows the percentage of cell cycle arrest for different compounds and the control sample

**Sample name**	**Sub G0** _ (% of the total population)_	**Singlet cell population** _(% of the parent)_	**G**_0_ **-G**_1 (% of singlet population)_	**S phase **_(% of singlet population)_	**M phase** _(% of singlet population)_
Control	18.22	77.72	54.09	7.18	36.57
Cisplatin	81.45	26.30	32.39	22.88	43.44
Bixin	52.45	58.46	31.65	13.20	53.48
Bixin+ Cisplatin	63.70	51.10	30.04	18.20	49.65
Crocin	53.69	69.03	49.11	14.01	36.12
Crocin+Cisplatin	89.26	24.39	20	33.04	30.87

## Conclusion

In this study, the DNA binding affinity with apocarotenoids and their combination with cisplatin was investigated using various techniques. UV-Visible spectroscopy studies suggest apocarotenoids and their combination show a non-covalent and non-intercalative mode of binding. Circular dichroism studies show minor changes in the amplitude of the ct-DNA spectra on the addition of the compounds and no significant change in the peak position which indicates groove binding interaction. DNA melt curve analysis presents a possible groove binding of the compounds observed through decreased fluorescence intensity. Viscosity studies demonstrated minor changes in the viscosity of ct-DNA solution with increasing concentration of apocarotenoids which suggests a groove binding mode of interaction. The *in vitro* analysis of the compounds with DNA proves that apocarotenoids and their combination have a considerable effect on DNA synthesis and could induce cell cycle arrest. Hence based on these observations, the apocarotenoids bixin and crocin do not intercalate but are very likely to bind to the groove of DNA. These carotenoids when combined with cisplatin seem to have synergistic effects as observed in cell cycle analysis. So, bixin and crocin can be potential candidates to treat cancer in combination with cisplatin or any other platinum-based drugs.

## Authors’ Contributions

JE M and S R designed the experiments; JE M performed experiments and collected data; JE M and S R discussed the results and strategy; JE M prepared the original draft; S R supervised and reviewed the draft. JE M and S R approved the version to be published.

## Funding

This article did not receive any specific grant from funding agencies in the public, commercial, or not-for-profit sectors.

## Conflicts of Interest

We have no conflicts of interest to disclose.
